# One-stage laparoscopy combined with resectoscope in the treatment of huge bladder diverticulum, multiple stones in diverticulum, multiple stones in bladder and benign prostatic hyperplasia: A case report

**DOI:** 10.3389/fmed.2022.1036222

**Published:** 2022-10-25

**Authors:** Wan Zhengqiang, Wang Yinglei, Li Cheng, Zheng Dongbing

**Affiliations:** ^1^Department of Urology, The Second Clinical Medical College of Binzhou Medical University, Shandong, China; ^2^The Second Ward of Urology, Yantai Affiliated Hospital of Binzhou Medical University, Yantai, Shandong, China

**Keywords:** Giant diverticulum of the bladder, diverticular calculi, bladder stones, benign prostatic hyperplasia, TURP, minimally invasive, one-stage surgery, case report

## Abstract

**Background:**

Bladder diverticulum is due to the abnormal arrangement of congenital bladder wall muscle fibers, weak limitations, combined with lower urinary tract obstruction, increased intravesical pressure, and protruding between the self-separated detrusor muscle bundles of the bladder wall. Giant bladder diverticulum refers to 10*8 cm or diverticulum over 150 ml in volume.

**Case summary:**

An 80-year-old male patient was admitted to our hospital on August 14, 2020, the preoperative diagnosis was: bladder diverticulum, bladder diverticulum calculi, multiple bladder stones and prostatic hyperplasia. On August 18, 2020, one-stage laparoscopic bladder diverticulectomy + diverticulum neck incision for stone removal + cystopuncture fistula + transurethral bladder stone removal + transurethral resection of the prostate (TURP) under general anesthesia. First, the bladder diverticulum was separated under laparoscopy, the diverticulum was incised, the diverticulum calculi were taken out, and then the diverticulum was completely removed, and the neck of the diverticulum was extended by 1.5 cm, and the large calculus of about 2.7*3.6 cm was completely removed, and then cystostomy + transurethral Bladder stone removal + TURP. There was no bleeding from the bladder suture during the operation. 200 ml of urine was drained from the extraperitoneal drainage tube, and 20 ml of urine was drained from the abdominal drainage tube during the operation, the urination is smooth, and the general condition can be discharged. The patient’s general condition is good after follow-up.

**Conclusion:**

One-stage laparoscopic treatment of bladder diverticulectomy + diverticulum neck incision for stone extraction + cystopuncture fistula + transurethral bladder stone extraction + TURP surgery. There is no report at home and abroad, which can provide diagnosis and treatment ideas and surgical methods for urological colleagues to deal with such diseases.

## Introduction

In patients with bladder diverticula, small diverticula that are asymptomatic do not require treatment. Large diverticula that cause recurrent urinary tract infections, dysuria, upper urinary tract damage or complications such as stones or tumor require surgical treatment. The first open bladder diverticulectomy in the world was performed by CZERNY (1) in 1897, Traditional open surgery to remove bladder diverticula is very invasive, with many post-operative complications and slow recovery, and is now being replaced by laparoscopic or robotic surgery (2–5). In 1992 Parra and Boulder (6) reported the first laparoscopic bladder diverticulectomy in the world, and in 2006, Xing et al. (7) reported laparoscopic transcatheter bladder diverticulectomy, which has been clinically proven to be feasible, with the advantages of less trauma, less blood loss, less postoperative pain, shorter hospital stay, wider operative field, aesthetic appearance and faster recovery (8).

The complete treatment of bladder diverticula is surgical removal of the diverticulum and simultaneous resolution of the cause of the obstruction (9). In 1996, Iselin et al. (10) were the first to report the sequential treatment of prostatic hyperplasia with bladder diverticula by electroporation and laparoscopic cystic diverticulectomy. In 2013, Li Haiping (11) reported extraperitoneal laparoscopic treatment of benign prostatic hyperplasia with bladder diverticulum. At present, 3 cases of benign prostatic hyperplasia and bladder diverticulum by laparoscopy combined with resectoscope (12–14) have been reported in China, while there have been no reports at home and abroad with huge bladder diverticulum, diverticulum calculi with BPH, and bladder calculi. In 2020, 1 case was treated in our hospital. One-stage laparoscopic bladder diverticulectomy + diverticulum neck incision stone removal + cystopuncture fistula + transurethral bladder stone removal + TURP, the treatment effect is definite, are reported as follows.

## Case report

### Introduction to medical records

The patient is an 80-year-old male, height: 166 cm, weight: 57 kg, nationality: Han nationality, occupation: freelancer. He was admitted to the hospital because of “progressive dysuria for more than 10 years, aggravating for more than 1 month.” About 10 years ago, the patient developed progressive dysuria without obvious incentives, such as waiting for urination, labored urination, thinning of the urination line, and incomplete urination. In 2015, he underwent transurethral resection of the prostate in our hospital and was discharged after his condition improved. About a month ago, the above symptoms aggravated, and the effect of oral “anti-inflammatory drugs” at home was not obvious. Now the patient came to our hospital for further diagnosis and treatment. After the outpatient examination, he was admitted to the Urology Department of our hospital with “prostatic hyperplasia and cystitis.” Since the onset of the disease, the patient has had no chills, no fever, normal diet and sleep, normal bowel movements, normal urination as described above, and no significant change in weight.

The patient’s previous physical condition was good. In 1970, the patient underwent subtotal gastrectomy for “peptic ulcer,” and the postoperative recovery was satisfactory. In 2015, the patient underwent transurethral resection of the prostate due to benign prostatic hyperplasia, and the postoperative recovery was satisfactory. The patient denied the history of hypertension, diabetes, coronary heart disease, viral hepatitis, tuberculosis infectious disease and close contact, denied any history of major trauma or blood transfusion and denied any history of drug or food allergies. Vaccination history unknown. Parents are not consanguineous, deny family history of disease and similar medical history.

The patient is in fair general condition, temperature: 36°C, pulse: 67 beats/min, respiration: 18 breaths/min, blood pressure: 169/97 mmHg. Abdomen is flat, no varices or scarring of the abdominal wall, no gastrointestinal pattern or peristaltic waves, soft to palpation, no pressure or rebound pain in the abdomen, liver and spleen are not palpable under the ribs, no percussion pain in the liver or kidney area, negative mobile turbid sounds, normal bowel sounds, no significant abnormalities in the bladder area. Rectal palpation: enlarged prostate, about 5*5*6 cm, tough, no hard nodules, no pressure pain, superficial central sulcus.

Ancillary examination: PSA assay: TPSA: 1.91 ng/ml, FPSA: 0.56 ng/ml. urine routine: BACT-M: 879/ul, WBC: 98.0/ul, BC: 2 +, BLD: 1 +, PRO: 4 +, NIT: 1 +, no abnormalities in the rest of the indicators. Coagulation analysis + D-dimer assay: DD: 0.63 mg/L, the remaining indicators were not abnormal. Liver function + cardiac enzyme assay: TP: 60.1 g/L, ALB: 34.9 g/L, CKMB: 33.2 U/L, no abnormalities in the remaining indicators. Urine culture + colony count: no bacterial growth in urine culture of the middle urine. Urology ultrasound shows prostate size is about 5*5*6 cm, the volume of diverticular urine was about 184 ml and the volume of residual urine in the bladder was about 125 ml. CT scan of the kidney and ureter and bladder + enhanced CT of the lower abdomen showed that the bladder was full, multiple stones were seen in the bladder, the larger one was about 2.7*3.6 cm; the bladder wall was thickened and strengthened, and multiple cystic pouch shadows were seen in part of the bladder wall protruding outwards with clear borders, the larger one was about 10.5*6.5 cm in size. The larger one is about 10.5*6.5 cm in size and contains stones. The prostate was enlarged and multiple foci of calcification were seen (Figure 2A). Diagnostic imaging: (1) bladder stones, cystitis and multiple diverticula formation. (2) prostatic hyperplasia and calcification. Cardiac ultrasound and cardiac function measurements: degenerative aortic lesion with a small amount of regurgitation, calcification of the posterior mitral leaflet annulus with a small amount of regurgitation, a small amount of tricuspid regurgitation, and left ventricular hypo-diastolic function. Pulmonary mediastinal CT scan: (1) multiple foci of fibrosis and microscopic sclerotic foci in the lungs, (2) double pulmonary emphysema, (3) calcification of the aortic and coronary artery walls. Pulmonary function measurements: normal ventilation, mildly reduced small airway function, normal ventilation reserve function. Electrocardiogram suggests: sinus rhythm; high voltage on the left ventricular surface and ST-T changes. Ultrasound of the liver, gallbladder, spleen and kidney: no significant abnormalities on ultrasound of the liver, gallbladder, spleen and kidney.

**FIGURE 1:**
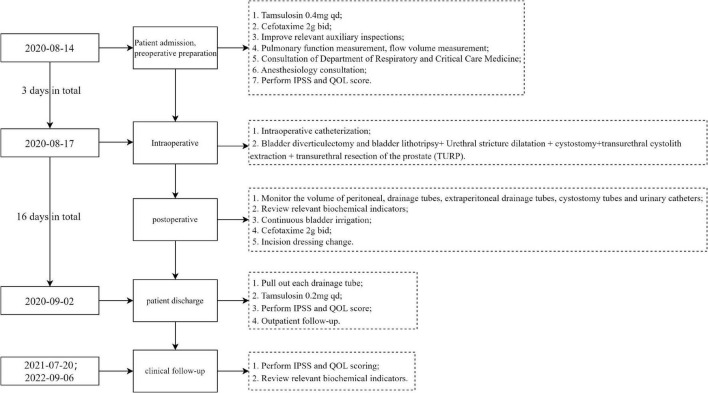
Diagnosis and treatment flow chart. This flowchart only includes the key events in the diagnosis and treatment process; IPSS, international prostate symptom score; QOL, quality of life.

**FIGURE 2:**
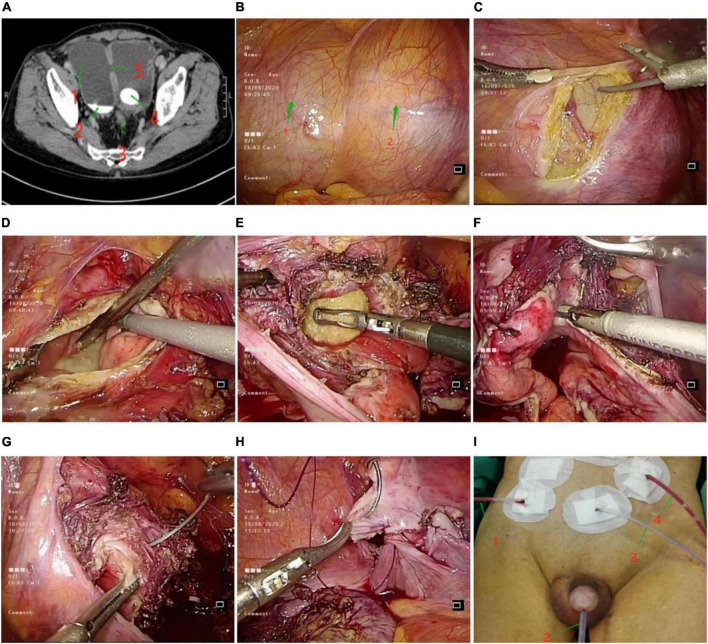
**(A)** Abdominal CT. 1: Bladder diverticulum; 2: Sedimentary stone; 3: Hyperplasia of prostate; 4: Bladder stone; 5: Orifice of bladder diverticulum. **(B)** a naturally full bladder under laparoscopy. 1: Naturally filled bladder under laparoscopy; 2: Naturally filled bladder diverticulum under the abdominal cavity. **(C)** Incision of bladder diverticulum with ultrasonic scalpel. **(D)** Removal of sediment-like stones in bladder diverticulum. **(E)** Enlarged neck of bladder diverticulum, huge bladder stone removal. **(F)** Ultrasound scalpel to remove bladder diverticulum tissue. **(G)** Full-thickness suture of the bladder wall using barbed sutures. **(H)** Suture the pelvic peritoneum using barbed sutures. **(I)** Four drainage tubes left after surgery. 1: Paravesical extraperitoneal drainage tube; 2: urinary catheter; 3: cystostomy tube; 4: abdominal drainage tube.

Combining the patient’s history, physical examination and ancillary examination, the initial diagnosis was: (i) prostatic hyperplasia; (ii) cystitis; (iii) bladder stones; (iv) bladder diverticulum; (v) bladder diverticulum stones; (vi) emphysema; (vii) post-gastrectomy for most of the stomach. The diagnosis of this patient is relatively easy, with typical clinical symptoms and imaging examinations. It should be noted that the clinical symptoms of bladder diverticula are generally not obvious. Larger bladder diverticula are prone to secondary infection, stone formation, and even tumor occurrence. The diagnosis is mainly based on imaging examinations. In addition, bladder stones in middle-aged and elderly people need to consider whether there is prostatic hyperplasia, because bladder stones are often secondary to urinary tract obstruction caused by prostatic hyperplasia. The patient’s diagnosis and treatment process were shown in Figure 1.

## Interventions

As the patient has cystitis as well as bladder stones, he has been advised to drink more water to dilute the urine and reduce bladder irritation, and also to use the antibiotic cefotaxime sodium (2g, bid) reasonably to fight infection and treat cystitis.

The patient is 80 years old and has emphysema. We have been considering whether he can tolerate anesthesia and surgery. In this regard, we actively take measures: (1) Further improve the pulmonary function test, which indicates that the lung function is normal; (2) We invite the department of respiratory and critical care and the department of anesthesiology for consultation. For emphysema, the latter consultation recommendations: (i) Considering the patient’s daily activities and pulmonary function (small airway function mild decline), the patient can tolerate anesthesia and surgery; (ii) The patient is old, and the cardiovascular and cerebrovascular accidents are higher than ordinary Crowd, fully communicate with family members before surgery. Special thanks to the professional advice of the two departments, combined with the consultation opinions, and communicated with the patients many times before the operation to eliminate the anxiety of the patients; (iii) Preoperative catheterization to reduce urinary retention in patients. The daily urine output of the patient is shown in Table 1; (iv) Continuously improve the preoperative examination and sign the informed consent for the operation, and fully prepare for the operation.

**TABLE 1:** Preoperative catheterization urine volume statistics (ml).

Day N of admission	16 h	2	3	4
Urinary catheter	600	1430	1500	800

h, hour.

In view of the huge bladder diverticulum, diverticulum with multiple stones bladder stones and BPH, according to the patient’s general condition, expert opinion and the surgical level of the attending surgeon, etc., it was agreed after many discussions and communications among the members of the medical team that: the patient was of advanced age and had emphysema, if open surgery was performed, it would be very traumatic and slow to recover, and bed resting would easily cause pneumonic pneumonia and coughing would easily cause poor healing of the incision, so minimally invasive surgery with less trauma and faster recovery Minimally invasive surgery with minimal trauma and quick recovery is the preferred treatment.

## Surgical ideas

In order to perform this minimally invasive surgery safely and smoothly, members of the medical team discussed and communicated many times, combined with the patient’s expectations for surgical treatment, we considered and addressed these 5 key questions: (i) Which surgical approach to choose? Is complete laparoscopic surgical treatment feasible? Considering that laparoscopic diverticulectomy + diverticulotomy for stone extraction followed by suturing of the bladder and then transurethral electrolysis of the prostate, there is a risk of intraoperative rupture of the suture and urinary fistula, so intraoperative cystocentesis is proposed to reduce the pressure in the bladder and reduce the risk of rupture of the bladder and urinary fistula. The patient had multiple stones in the bladder, and for large stones (about 2.7*3.6 cm), we considered that if the anterior bladder wall was cut to remove the stones, it would be more traumatic and also increase the risk of postoperative vesicourethral fistula, so it was not the preferred way to remove the stones. The large bladder stones can be removed easily and minimally invasively, and the remaining multiple small stones can be flushed out by irrigation fluid during TURP. (ii) Can the surgeon be competent for this complex surgical approach? The surgeon has previous experience in laparoscopic cystic diverticulectomy, open cystic diverticulectomy, open cystotomy and stone extraction, transurethral bladder stone lithotripsy and transurethral electrodesiccation of the prostate, and the surgeon is highly competent to perform this procedure. (iii) Should I choose the first or second stage of surgery? The patient was 80 years old and had emphysema. The second-stage surgery was very traumatic and the patient’s recovery was slow and there were many post-operative complications, whereas the first-stage minimally invasive treatment was less traumatic and the patient recovered quickly, and the patient had acceptable cardiopulmonary function and general condition and could tolerate anaesthesia and surgery. (vi) Alternate surgical plan? As this procedure is the first of its kind at home and abroad, there is not much literature to refer to, so we cannot fully consider the intraoperative situation, nor can we specify the duration of the operation. To ensure surgical safety, we consider that if the laparoscopic cystectomy + diverticulotomy to remove the stone takes longer, then we will consider performing transurethral resection of the prostate in the second stage. (v) The patient is 80 years old and in a hypercoagulable state postoperatively, how can we avoid venous thrombosis of the patient’s lower limbs? To avoid lower limb vein thrombosis, we consider laparoscopic bladder diverticulectomy + diverticulotomy for stone extraction in the flat position, and then in the lithotomy position for transurethral bladder stone extraction + TURP. Although the change in position requires re-sterilisation of the sheet, it effectively reduces the risk of lower limb venous thrombosis. The patient’s coagulation analysis + D-dimer is regularly reviewed postoperatively and low molecular heparin anticoagulation is given if necessary to prevent lower limb venous thrombosis. Please refer to the surgical record for specific intraoperative conditions.

## Surgical records

### Bladder diverticulectomy and bladder lithotripsy

Under general anesthesia, the patient was placed in a supine position of 25 degrees with head down and feet elevated, and the skin of the surgical field was routinely disinfected and draped. A 2 cm arc-shaped incision was made at the lower edge of the umbilicus, pneumoperitoneum was created with a pneumoperitoneum needle, and CO_2_ gas was injected to maintain 12 mmHg. The bladder diverticulum was separated under laparoscopy (Figure 2C), the diverticulum was incised, and sand-like stones were seen in the diverticulum (Figure 2D), a suction device was used to suck out the sediment-like stones, and the larger stone separation forceps were taken out. Enlarge the neck of the bladder diverticulum, see a calculus of about 2.7*3.6 cm in the bladder (Figure 2E), which is clamped out with grasping forceps. ultrasound scalpel to remove all bladder diverticulum tissue (Figure 2F). The full-thickness bladder wall was sutured with 2.0 barbed thread (Figure 2G), the seromuscular layer was embedded, the pelvic peritoneum was sutured (Figure 2H), and an extraperitoneal drainage tube beside the bladder was indwelled. No obvious exudation was observed after intravesical infusion. There was no active bleeding in the operative field, and a drainage tube was placed in the abdominal cavity. See Figures 2B–H for the entire surgical procedure.

### Urethral stricture dilatation + cystostomy + transurethral cystolith extraction + transurethral resection of the prostate

The patient was taken in the lithotomy position, routinely disinfected, draped, and connected to resectoscope instruments and wires. The patient’s urethral stenosis was seen during the endoscopy. After urethral dilatation was given, the endoscopy was continued, and it was seen that the prostatic hyperplasia on both sides of the prostate was obvious, and there were multiple bladder diverticula in the bladder. Small bladder stones were seen in some diverticulum, the larger ones were about 1.0*0.8 cm, and normal saline was injected into the bladder until the bladder was filled and expanded; a small incision was made with the two transverse fingers of the upper pubic bone in the middle of the lower abdomen, and the length was about 0.5 cm, cystopuncture fistula The device was punctured under the direct vision of the resectoscope, and the F16 double-lumen urinary catheter was indwelled through the fistula device, and the skin was sutured and fixed. After the bladder stones were flushed out by intravesical injection of water, the power of electrocution was set to 300 w, and the electrocoagulation was set to 80 w. The hyperplasia of the prostate tissue was electrocuted to reach the surgical capsule, and the distal end to the fine fu. Care should be taken to protect the sphincter and electrocoagulate the wound to stop bleeding. The prostate tissue and blood clots are removed and sent to pathology.

### Surgical results and post-operative follow up

The total operation time was 4 h, the operation time of bladder diverticulectomy was 58 min, the total blood loss during the operation was 20 ml, the catheter was pulled out 14 days after the operation, and the patient was hospitalized for a total of 19 days. The ureter, bowel, and pelvic vessels were not damaged during the operation, and there were no complications such as prostate capsule perforation, resection syndrome, and urethral sphincter injury. The most common complications of this operation are urine leakage and urine extravasation. The exudate or exudate will contaminate the abdominal cavity and seriously affect the healing of the bladder incision. To avoid this, various measures have been taken during the operation. See the discussion section for details. In order to monitor the occurrence of this situation after surgery, the drainage volume of each drainage tube of the patient has been recorded after surgery (Figure 2I). The statistics are as follows (Table 2).

**TABLE 2:** Post-operative abdominal drainage tube drainage statistics (ml).

N day after surgery	11 h	2	3	4	5	7	8	9	10	11	12
Abdominal drainage tube	20	5	x								
Extraperitoneal drainage tube	20	8	5	x							
Cystostomy tube	40	1650	1800	2025	2300	3000	2950	1400	1100	1050	800
Urinary catheter	300	1200	1450	1200	1050	550	1000	1500	1300	1450	1600

x, indicates drainage tube has been removed; h, hour.

From the drainage volume of each drainage tube after operation, it can be seen that each drainage tube is unobstructed, the peritoneal exudate is less, the bladder is sutured tightly, and no urine leakage or extravasation occurred. No significant abnormalities were seen in the routine blood tests repeated on the 3rd and 7th postoperative days. Histopathological examination of the prostate: benign prostatic hyperplasia; chronic prostatitis. Histopathological examination of the bladder diverticulum: more acute and chronic inflammatory cell infiltration with lymphocytic infiltration was seen in the mucosal tissue sent for examination, which was consistent with diverticular changes. The patient was able to pass urine without complications such as urinary incontinence and urethral stricture, and the surgical expectations were achieved.

Two-year follow-up after operation, the patient recovered well after operation, the symptoms of urinary tract obstruction were significantly relieved, there was no urinary incontinence, urinary fistula complications, and no bladder diverticulum and stone recurrence.

## Discussion

### Pathogenesis

Bladder diverticulum is formed due to the abnormal arrangement of the muscle fibers of the congenital bladder wall, the weakness of the limitation, the obstruction of the lower urinary tract, the increase of the intravesical pressure, and the protrusion between the detrusor muscle bundles of the bladder wall self-separation (11). Bladder diverticulum can be divided into congenital and acquired. Congenital bladder diverticulum, also known as true diverticulum, is mostly single. The wall of the diverticulum contains a muscular layer, which may be due to the local dysplasia or lack of development of the detrusor muscle of the bladder, which causes the bladder mucosa to protrude outward from the detrusor muscle fibers, resulting in bladder diverticulum. Acquired bladder diverticulum is formed by the long-term increase of intravesical pressure and the protrusion between the detrusor muscle bundles separated from the bladder wall. Mainly located in the lateral ureter and the posterior wall of the bladder, the common clinical diverticulum is acquired diverticulum. Urine retention within the diverticulum can lead to bladder infections and stones, occasionally combined with diverticular tumor (12). In this case, the patient had a large diverticulum secondary to BPH, combined with diverticular calculi and multiple bladder stones.

### Current status of treatment

It is very unlikely that a bladder diverticulum will require surgical removal, but surgery is required in the following cases: (i) the ureteral opening is adjacent to the diverticulum or is located within the diverticulum and there is vesicoureteral reflux; (ii) the diverticulum is located at the base of the bladder and causes obstruction to the bladder outlet; (iii) there is a combination of infection; (vi) there is a diverticulum with stones or tumor (13); (v) diverticula with stones or tumor.

Current status of treatment: open surgical resection is the traditional treatment of choice for bladder diverticula. Minimally invasive surgery for bladder diverticulum has the advantages of small wound, low complications, quick postoperative recovery and short hospital stay. At present, conventional laparoscopic and robot-assisted laparoscopic surgery are the main minimally invasive surgical methods for the treatment of bladder diverticulum (4). (i) Localization of bladder diverticula. Laparoscopic bladder diverticulectomy, finding the diverticulum is an extremely critical step of the procedure, foreign scholars use the light source of the cystoscope to irradiate the diverticulum (15) or by placing a balloon urethral tube inside the diverticulum, etc. There is also the use of electronic cystoscopy to guide bladder diverticula, and it is believed that the combination of flexible cystoscopy and robotic surgery is helpful for the removal of bladder diverticulum. There is also the use of electronic cystoscopy to guide bladder diverticula, and it is believed that the combination of flexible cystoscopy and robotic surgery is helpful for the removal of bladder diverticulum (16). In China, it has also been reported that (17) the use of intra-diverticular placement of balloon urinary catheters to find diverticula. (ii) Prevent damage to the ureter. Another key to this procedure is to correctly determine the anatomical relationship between the diverticulum and the ureter. The most common method is to place a ureteral stent tube or a double J tube in the ureter before surgery to prevent ureteral injury, so that even if the ureter is injured, it can be detected and treated early, thus avoiding the occurrence of postoperative urinary leakage (18). Xing et al. (7) also proposed electrocoagulation at the neck of the diverticulum to improve the accuracy of diverticulectomy and thus avoid inadvertent ureteral injury. (iii) Bladder stone removal method. With the development of intracavitary urology in recent years, the current methods of bladder stone extraction are diversified, (19). The following methods are available: small incision cystotomy in the lower abdomen, small incision cystotomy in the lower abdomen (20). At present, the most common methods are endoscopic mechanical lithotripsy and ureteroscopic Holmium laser lithotripsy (21). (vi) Treatment of benign prostatic hyperplasia and its complications. Elderly BPH complicated with bladder stones not only affects the patient’s life, but can even induce bladder cancer (22). When BPH occurs, the total volume of the prostate increases, which affects the anatomy of the prostate, causing the flow of urine to impact the urethral wall, resulting in a stress response, aggravating hyperplasia, and increasing the risk of bladder stones (23). For bladder diverticulum with benign prostatic hyperplasia, there are two methods of staged management: one is to remove the bladder outlet obstruction by TURP first and then perform bladder diverticulectomy in the second stage; the other is to treat the bladder diverticulum first and then perform TURP in the second stage to remove the bladder outlet obstruction, which has a longer hospital stay, more bleeding, more trauma, longer recovery time and requires a second operation (24). In recent years, with the advancement of technology and equipment, the one-stage treatment of BPH and bladder diverticulum has been gradually developed and improved. In 2013, Li et al. (25) reported the extraperitoneal laparoscopic treatment of benign prostatic hyperplasia with bladder diverticulum, and believed that the extraperitoneal laparoscopic treatment of prostatic hyperplasia with bladder diverticulum was feasible and effective. Similar efficacy to surgery, but also has the advantages of less pain and faster recovery for patients undergoing minimally invasive surgery. This year, robotic surgery has also begun to be used to treat BPH. Singh et al. (26) reported a patient with a giant prostate who underwent robotic-assisted laparoscopic prostatectomy.

### Innovations in this case

(I) To locate the bladder diverticulum, in this case no catheter was left in place preoperatively and urine was used to naturally fill the bladder and diverticulum to find the diverticulum and bladder (Figure 2B) for two reasons. (i) The patient had a large bladder diverticulum (approximately 10.5*6.5 cm). (ii) The size and location of the diverticulum and its relationship to the ureter have been clarified by preoperative enhanced CT (Figure 2A). Therefore, for larger bladder diverticula, this method can be considered. It is reliable for finding diverticula, simple to operate, reduces the separation of the peritoneum, facilitates the restoration of peritoneal integrity after resection, and basically achieves anatomical repositioning of the peritoneum after surgery, and reduces the interference of extravasated urine with the intestinal canal and visceral organs.

(II) To prevent ureteral injury, a ureteral stent was not placed in the ureter preoperatively in this case, nor was a double J tube left in place, for three reasons. (i) The anatomical position of the bladder diverticulum and the ureteral orifice is far away from the preoperative examination. (ii) The operator had previously performed this type of procedure and was technically proficient and the possibility of intraoperative damage to the ureter was unlikely. (iii) There is a high chance of retrograde infection from the retained double J tube and the need to operate again in order to remove it is more traumatic. Therefore, in clinical practice, if the location of the ureteral orifice and the bladder diverticulum are unclear or if they are close to each other, we can consider placing a ureteral stent or leaving a double J tube in place to prevent ureteral injury.

(III) New Ideas for Bladder Stone Removal, for patients with bladder diverticula combined with huge bladder stone, can be considered to extend the diverticulum neck opening, through the bladder diverticulum neck opening this pathological channel to remove huge bladder stone in the bladder. The author believes that this method can be considered in patients with bladder diverticula and bladder stones, without the need for incision of the anterior bladder wall to retrieve the stone and reduce injury.

(IV) Three measures to prevent urine leakage and extravasation. postoperative urinary leakage is the most common complication of laparoscopic bladder diverticulectomy, which is mostly caused by poor intraoperative exposure and inaccurate suture of the bladder wall (27). The following methods can be used: (i) The bladder is sutured with full-thickness suture + seromuscular embedding suture to facilitate the healing of the bladder wound. After suture, the bladder water injection test is performed to check whether the bladder wound is closed tightly; (ii) Intraoperative cystocentesis, reduced bladder pressure during prostatectomy; (iii) The pelvic floor peritoneum is completely sutured to achieve its anatomical reduction, restore the continuity of the pelvic floor peritoneum, and indwell one extraperitoneal drainage tube next to the bladder and one abdominal cavity drainage tube. The tube can also drain the urine in time to avoid its contamination of the abdominal cavity. One extraperitoneal drainage tube next to the bladder and one abdominal cavity drainage tube are indwelled. Drainage tubes and abdominal drainage tubes can also drain urine in time to avoid contamination of the abdominal cavity.

(V) Cystotomy followed by TURP: Some scholars believe that when TURP is performed, due to the lack of treatment of BPH during the diverticulectomy, the residual urine increases and the pressure in the bladder rises, making it difficult for the incision at the diverticulectomy site to heal and even rupture, leading to extravasation (8). Therefore, cystostomy can be performed before TURP to reduce the pressure in the bladder, prevent urine leakage and extravasation from the suture, and promote wound healing.

In conclusion, the one-stage laparoscopic combined with electrodesiccoscopic management of giant bladder diverticula, diverticula with multiple stones and bladder with multiple stones and prostatic hyperplasia, compared with the previous surgical methods for dealing with such diseases, it has been greatly improved and innovative, and its surgical effect and prognosis are outstanding.

### Disadvantages of this surgical approach

Although this surgical method has many advantages over traditional surgical methods, it also has some disadvantages. For example, it has high requirements on the physical condition of the patient, high technical requirements on the operator, and requires a long preoperative preparation.

## Ethics statement

The studies involving human participants were reviewed and approved by Ethics Committee of Binzhou Medical University. The patients/participants provided their written informed consent to participate in this study.

## Author contributions

WZ designed and conducted the whole research. LC collected patient clinical information and conducted postoperative follow-up. WZ completed the data analysis and drafted the manuscript. WY revised and finalized the manuscript. All authors contributed to the article and approved the submitted version.
